# Enhancement of Curcumin Bioavailability Using Nanocellulose Reinforced Chitosan Hydrogel

**DOI:** 10.3390/polym9020064

**Published:** 2017-02-15

**Authors:** Thennakoon M. Sampath Udeni Gunathilake, Yern Chee Ching, Cheng Hock Chuah

**Affiliations:** 1 Department of Chemical Engineering, Faculty of Engineering, University of Malaya, 50603 Kuala Lumpur, Malaysia; sgunathilake@yahoo.com; 2 Department of Chemistry, Faculty of Science, University of Malaya, 50603 Kuala Lumpur, Malaysia; chchuah@um.edu.my

**Keywords:** biodegradable, chitosan, curcumin, nanocellulose, drug delivery, bioavailability

## Abstract

A unique biodegradable, superporous, swellable and pH sensitive nanocellulose reinforced chitosan hydrogel with dynamic mechanical properties was prepared for oral administration of curcumin. Curcumin, a less water-soluble drug was used due to the fact that the fast swellable, superporous hydrogel could release a water-insoluble drug to a great extent. CO_2_ gas foaming was used to fabricate hydrogel as it eradicates using organic solvents. Field emission scanning electron microscope images revealed that the pore size significantly increased with the formation of widely interconnected porous structure in gas foamed hydrogels. The maximum compression of pure chitosan hydrogel was 25.9 ± 1 kPa and it increased to 38.4 ± 1 kPa with the introduction of 0.5% cellulose nanocrystals. In vitro degradation of hydrogels was found dependent on the swelling ratio and the amount of CNC of the hydrogel. All the hydrogels showed maximum swelling ratios greater than 300%. The 0.5% CNC-chitosan hydrogel showed the highest swelling ratio of 438% ± 11%. FTIR spectrum indicated that there is no interaction between drug and ingredients present in hydrogels. The drug release occurred in non-Fickian (anomalous) manner in simulated gastric medium. The drug release profiles of hydrogels are consistent with the data obtained from the swelling studies. After gas foaming of the hydrogel, the drug loading efficiency increased from 41% ± 2.4% to 50% ± 2.0% and release increased from 0.74 to 1.06 mg/L. The drug release data showed good fitting to Ritger-Peppas model. Moreover, the results revealed that the drug maintained its chemical activity after in vitro release. According to the results of this study, CNC reinforced chitosan hydrogel can be suggested to improve the bioavailability of curcumin for the absorption from stomach and upper intestinal tract.

## 1. Introduction

Hydrogels are highly swollen, hydrophilic, three-dimensional (3D) polymeric networks capable of absorbing large amounts of water without dissolving in water or aqueous solvent, yet are insoluble due to the presence of cross-links, entanglements, or crystalline regions [1]. Hydrogel can be formed by natural, synthetic or their hybrid polymers. Recently, attention has been focused on producing hydrogels using materials that are non-toxic and biocompatible in nature. Considering not only safety, but also biocompatibility and biodegradability, biopolymer-based hydrogels have been drawing increasing interest and attention as excellent candidates for biomedical applications, such as drug delivery, wound dressings, and tissue engineering matrices [2].

Biocompatible and biodegradable hydrogels are very important biomaterials used in drug delivery systems, which gained increasing attention of researchers. Chitosan is a highly swellable, pH responsive and biocompatible polymer which can be used to deliver drugs to specific sites of gastrointestinal tract. The pH-sensitive swelling and releasing behavior of hydrogels are useful for the design of oral drug delivery carrier. Chitosan is a linear ubiquitous polysaccharide and heteropolymer consist of glucosamine and *N*-acetyl-glucosamine units [3]. Amino groups of chitosan have a p*K*a value of around 6.5. At pH below this p*K*a value, the amino groups of chitosan get ionized and positively charged –NH_3_^+^ groups are distributed among hydrogel network. This leads to the repulsion of polymer chains within the hydrogel and allowing more water intake into the hydrogel network [4]. Referring to our previous study [5], chitosan hydrogel also exhibited a high swelling ratio in acidic medium than alkaline conditions. Cationic nature of chitosan in acidic medium increases the retention of the hydrogel at the site of application and readily binds to negatively charged surfaces such as mucosal membranes. Mucoadhesive ability could result in formulations containing chitosan being retained in the mucosal surface such as gastric mucosa due to the acidic environment of the stomach. Due to the high degree of swelling and mucoadhesive properties in acidic medium, chitosan-based hydrogels have been used as a carrier for stomach—specific drug delivery systems [6,7,8,9,10,11].

Adequate mechanical strength is desirable for hydrogels as drug delivery systems [12,13]. Improved mechanical strength can withstand the pressure during gastric contraction and prolong the gastric retention time [14]. Hydrogels with less mechanical strength can become fragmented after repetitive gastric contractions [15,16]. Recently, nanomaterials have been used as a reinforcing agent to improve the mechanical strength and stability of polymer hydrogels [17,18]. CNCs have received much attention as reinforcing agent because of their properties such as large surface area, high mechanical strength, low density, non-toxic nature, high aspect ratio, biocompatibility and biodegradability [18,19,20,21,22,23]. In our previous study [4], the compression strength of the chitosan hydrogel increased with physical reinforcement of cellulose nanocrystals. The maximum compression of chitosan hydrogel increased from 38.4 ± 1 kPa to 50.8 ± 3 kPa, with increasing CNC content from 0.5% to 2.5%. Nanocellulose reinforced chitosan hydrogel forms a semi-interpenetrating polymer network (semi-IPN) by diffusion of linear polymer chains into a preformed polymer network. Semi-IPN improves the mechanical properties and controls the swelling behavior of hydrogel [5,24].

Curcumin (diferuloylmethane), a natural polyphenolic nutraceutical, is a major component of turmeric (*Curcuma longa*) that has been associated with antioxidant, anticancer, anti-inflammatory, antiviral, and antibacterial activities as indicated by over 6000 citations and over one hundred clinical studies [25]. Recent studies showed that curcumin can block ethanol, indomethacin, stress-induced gastric ulcer and can prevent pylorus-ligation-induced acid secretion [26]. *Helicobacter pylori* (*H. pylori*) a gram-negative bacteria, is reported as etiologic factor in the development of the chronic gastritis, ulcers and gastric adenocarcinoma. Many studies highlighted the potential of curcumin as a promising antibacterial agent having property to restore and repair the gastric damage caused by *H. pylori* infection. The poor solubility and low bioavailability of curcumin have been highlighted as the major problem that can lead to loss of local therapeutic action in the stomach [27]. However, many attempts have been made to improve the pharmacotherapy of the stomach through local drug release, leading to high drug concentration at the gastric mucosa, making it possible to treat stomach and duodenal ulcer, gastritis and esophagitis [10,28,29,30,31,32,33].

CNC/chitosan hydrogels have been used for various drug delivery applications. Previous studies [34,35] had reported the use of CNC/chitosan hydrogels as potential drug carrier for procaine hydrochloride and formation of polyelectrolyte-macroion complexes. However, nanocellulose reinforced chitosan hydrogel is not yet investigated or reported for its application as drug delivery system for curcumin. Objective of this study is to improve the bioavailability of less water-soluble curcumin for the absorption from stomach and upper intestinal tract. In acidic medium, the protonated amino groups of chitosan will interact with sialic acid (*N*-acetylneuraminic) in gastric mucus by electrostatic interaction thus improve the gastric residence time and retaining the drug at the absorption site for a prolonged period. A large extent of chitosan matrix swelling is found to occur in acidic medium, hence the drug molecules are expected to diffuse extensively through the swollen gel in to the exterior medium at gastric pH levels. Due to the fast swelling property the super porous hydrogels are widely used for delivery of poorly water-soluble drugs. Gas foaming is used to fabricate superporous hydrogel with large and widely interconnected pore structures. To sum-up, the mucoadhesiveness, stimuli sensitivity and faster swellability will make the hydrogel a suitable candidate for stomach specific drug delivery systems.

## 2. Materials and Methods

### 2.1. Materials

Chitosan medium molecular weight (viscosity 200–500 cP, 0.5% acetic acid at 20 °C), acetic acid glacial grade AR, methanol, hydrochloric acid, sodium chloride and sulfuric acid were purchased from Friendemann Schmidt Chemicals (Parkwood, Australia). Glutaraldehyde 25% was obtained from Thermo Fisher Scientific Inc. (Victoria, Australia). The drug curcumin was provided by Himedia Laboratories Pvt Ltd. (Mumbai, India). Microcrystalline cellulose and phosphate-buffered saline was obtained from R&M chemicals (Essex, UK). All chemicals were used as obtained.

### 2.2. Preparation of CNC–Chitosan Hydrogel

CNCs were prepared from microcrystalline by sulfuric acid hydrolysis method reported in our previous study [36,37,38]. Chitosan was dissolved in 5% (*v*/*v*) aqueous acetic acid solution at room temperature and left overnight in the shaker with the rotation rate of 250 rpm to prepare a 2% (*w*/*v*) chitosan solution (High concentration of acetic acid induces lower viscosity and hence easier to dispersion of nanocrystals) [39,40]. The solution was then filtered through the filter paper to remove any insoluble matters. CNCs were homogenized using ultrasonic treatment for 10 min to obtain stable and uniformly distributed nanocrystals. To prepare chitosan hydrogel and CNC–chitosan hydrogel, CNCs in different concentrations were added (0%, 0.5%, 1%, 1.5%, 2%, 2.5%) to 2% (*w*/*v*) chitosan solution and stirred (250 rpm) for one hour. After that 0.2% (*v*/*v*) of glutaraldehyde (in the final mixture) was mixed with CNC–chitosan solution and stirred (350 rpm) for 1 min at room temperature. The mixture was then poured into the mold and allowed the hydrogel to solidify at room temperature for 24 h. Finally, hydrogels were rinsed several times with distilled water to remove any unreacted polymer or chemicals.

### 2.3. Equilibrium Swelling Study

The swelling ratios were measured by the immersion of hydrogels in distilled water at 37 °C. Before the swelling test, hydrogels were cut into disk shape pieces and dried in room temperature until they reached constant weight. Dried hydrogels were weighed before immersion into the distilled water. The weight of the swelled hydrogels after immersion in the distilled water was recorded at predetermined time intervals over 16 h. The swelling ratio of the hydrogels was calculated using Equation (1).

(1) $$\begin{array}{c} \mathrm{Swelling} \end{array}\begin{array}{c} \mathrm{ratio} \end{array}\begin{array}{c} \left(\%\right) \end{array}=\frac{W_{1}-W_{0}}{W_{0}}\begin{array}{c} \times \end{array}\begin{array}{c} 100 \end{array}$$

where *W*_1_ is the weight of swollen hydrogel and *W*_0_ is the initial dry weight of the hydrogel.

### 2.4. CO_2_ Gas Foaming of Hydrogels

A 0.5% CNC–chitosan composition was used for the high pressure CO_2_ gas foaming process as it showed the highest swelling ratio from equilibrium swelling test. After mixing with the chitosan solution with glutaraldehyde and 0.5% CNC as described above, the mixture was poured in to the mold and placed inside the gas foaming apparatus. After that the apparatus was gradually pressurized with CO_2_ to predetermined pressure (10, 30 and 50 bar). The pressure was maintained to allow for CO_2_ saturation and chitosan crosslinking for 48 h. The system was then depressurized at 1 bar/min, resulting in the generation of numerous gas bubbles which induce gas foaming. Swelling tests, drug loading and releasing tests were done for 0.5% CNC–chitosan hydrogel prepared at 10, 30 and 50 bar pressure and compared with the results of the hydrogel prepared at atmospheric condition.

### 2.5. Drug Loading Efficiency

Curcumin was entrapped into hydrogels by swelling equilibrium method. Disk shaped hydrogels were immersed in 30 mg/L drug solution for 24 h at room temperature. Curcumin loaded hydrogels were removed from drug solution and rinsed with distilled water and washing was collected. Curcumin concentration remaining in the solution was determined by UV-2600, UV-Vis spectrophotometer (Shimadzu, Kyoto, Japan) at 427 nm. The experiments were repeated three times and average values were taken. The drug encapsulation efficiency was calculated using Equation (2).

(2) $$\mathrm{Encapsulation}\begin{array}{c} \mathrm{efficiency}\begin{array}{c} \%\begin{array}{c} =\begin{array}{c} \frac{\mathrm{Amount}\begin{array}{c} \mathrm{of}\begin{array}{c} \mathrm{curcum}\begin{array}{c} \mathrm{in}\begin{array}{c} \mathrm{the}\begin{array}{c} \mathrm{hydrogel} \end{array} \end{array} \end{array} \end{array} \end{array}}{\mathrm{Initial}\begin{array}{c} \mathrm{drug}\begin{array}{c} \mathrm{amount}\begin{array}{c} \mathrm{in}\begin{array}{c} \mathrm{the}\begin{array}{c} \mathrm{solution} \end{array} \end{array} \end{array} \end{array} \end{array}} \end{array} \end{array} \end{array} \end{array}\begin{array}{c} \times \begin{array}{c} 100 \end{array} \end{array}$$

### 2.6. In Vitro Drug Release

In vitro drug release from hydrogel networks with different drug loading contents, was investigated in simulated gastric condition (prepared by dissolving 2 g NaCl in 7.0 mL HCl and water up to 1000 mL) at 37 °C. In order to study the release, 3 mL solution containing released drug was removed at predetermined time intervals and returned it back to the solution after the analysis. The concentration of released curcumin was measured at 427 nm using UV-Vis spectrophotometer (UV-2600, Shimadzu, Japan). The experiments were performed triplicates and average values were taken.

### 2.7. FTIR Analysis

FTIR studies of curcumin, hydrogel and curcumin loaded hydrogel were carried out using PerkinElmer spectrum 400 FTIR spectrometer (Shimadzu, Kyoto, Japan) over the range 3000–500 cm^–1^.

### 2.8. Morphology Studies

The morphology of the gas foamed hydrogel (at 50 bar) and hydrogels prepared at atmospheric condition was examined using (FE-SEM, SU8220) field emission scanning electron microscope (Hitachi, Tokyo, Japan). Hydrogels were freeze dried using freeze dryer to remove water without disturbing the morphology. After this, the hydrogels were coated with gold in order to prevent the charging effects at an accelerating voltage of 5 kV.

### 2.9. Mechanical Testing

The mechanical properties of hydrogels were investigated by compression test using a (Shimadzu, AGS-X) universal/tensile tester (Shimadzu, Kyoto, Japan). The hydrogels were cut in to disk shapes (thickness (12 mm) and diameter (18 mm)) and allowed to equilibrate in pH 7.4 buffer solutions for 30 h. During the compressive strength tests, stress and strain responses were monitored under a load of 500 N and at a rate of 0.5 mm/min. Strain and stress were recorded using Trapezium Lite X software until maximum breaking strength was approached. Five replicates were tested to get the average and standard deviation.

### 2.10. In Vitro Degradation

In vitro degradation studies of hydrogels were carried out in PBS solution for six weeks. The dried gels were kept in 50 mL of PBS. The PBS was renewed every week. At predetermined time intervals, samples were taken out, washed, blotted using filter paper and dried to constant weight at 45 °C. Dry weight of the sample was measured and weight loss was determined using Equation (3).

(3) $$\mathrm{Weight}\begin{array}{c} \;\mathrm{loss}\begin{array}{c} \%\begin{array}{c} =\begin{array}{c} \frac{W_{0}\begin{array}{c} -\begin{array}{c} W_{t} \end{array} \end{array}}{W_{0}} \end{array} \end{array} \end{array} \end{array}\begin{array}{c} \times \begin{array}{c} 100 \end{array} \end{array}$$

*W*_0_ is the dry weight of the hydrogel taken initially and *W*_t_ is the dry weight at time *t*.

## 3. Results and Discussion

### 3.1. Mechanical Properties

Adequate mechanical strength is desirable for hydrogels as drug delivery systems. Improved mechanical strength can withstand the pressure during gastric contraction and prolong the gastric retention time. Hydrogels with less mechanical strength can become fragmented after repetitive gastric contractions [15,16]. Unmodified chitosan hydrogels exhibit relatively low mechanical strength uncontrollable porosity and degradability particularly in acidic aqueous solutions [41]. Thus many efforts have been made on modification of chitosan facilitated by its hydroxyl and amino groups [42]. Mechanical strength, can be improved by covalent crosslinking using chemical crosslinkers such as glutaraldehyde (GA), oxalic acid, formaldehyde, glyoxal, and genipine [43]. Previous studies revealed that the higher crosslinker concentrations are favorable from the mechanical stability point of view, but at the same time the decrease in porosity may cause lower drug release rates by diffusion through the porous media [44]. In general, a high concentration of crosslinking agent is required to improve the mechanical strength of a hydrogel [16]. In this research, we were able to improve the mechanical properties of chitosan hydrogel by physical reinforcement of CNC at a constant crosslinker concentration. Glutaraldehyde (0.2% (*v*/*v*)) was used to prepare both pure chitosan and 0.5% CNC reinforced chitosan hydrogel. Nanocellulose reinforced chitosan hydrogel forms a semi-interpenetrating polymer network (semi-IPN) via the diffusion of linear polymer chains into a preformed polymer network. A semi-IPN structure improves the mechanical properties of a hydrogel and controls its swelling behavior [30].

Several mechanisms are responsible for the interface reinforcement of polymer matrix by nanoparticles. The interaction between nanoparticles and the polymer matrix could form special microstructures such as finer scale lamellar structure result in improved mechanical properties; nanoparticles could effectively enhance their interaction with the matrix through chemical bonds (for instance, increase the crosslinking densities) or increase the physical interactions between polymer chains of the matrix. In this manner, nanoparticles can strongly influence to complement the poor mechanical and tribological performances of some polymer matrices [31].

In our previous study, the maximum compression of CNC–chitosan hydrogel increased from 25.9 ± 1 kPa to 50.8 ± 3 kPa with increasing CNC content ranging from 0% to 2.5%. In addition, the maximum compression did not increase significantly with the addition of over 2.5% CNCs to the chitosan hydrogel [4]. The results of previous study indicated a decrease in swelling ratios with increasing the CNC content of chitosan hydrogel. Within the CNC reinforced hydrogels, 0.5% CNC–chitosan hydrogel indicated the highest swelling ratio in distilled water. Therefore, 0.5% CNC–chitosan hydrogel was used for the gas foaming process and for the investigation of mechanical properties.

**Figure 1:**
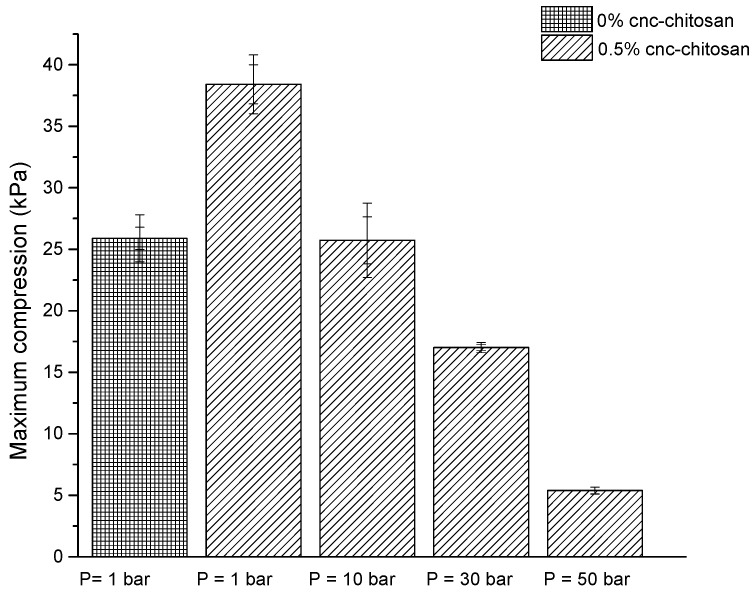
Maximum compression of hydrogels formed at different pressure conditions.

As shown in Figure 1, the maximum compression of pure chitosan hydrogel was 25.9 ± 1 kPa and it increased to 38.4 ± 1 kPa with the introduction of 0.5% cellulose nanocrystals. The maximum compression of 0.5% CNC–chitosan hydrogel produced at atmospheric condition was 38.4 ± 1 kPa and it decreased with increasing the pressure of gas foaming process. This value for hydrogels produced at 10, 30 and 50 bar was 25.7 ± 2, 17.0 ± 0.2 and 5.4 ± 0.2 kPa, respectively. The decrease in maximum compression is due to the increase of pore size and the pore interconnectivity of CNC–chitosan hydrogel produced at gas foaming process. Several studies have also reported that the mechanical strength of hydrogels and scaffolds decreases with increase of the pore size and the pore interconnectivity [29,32,45]. Ji et al. [46] reported that the compressive modulus of glutaraldehyde crosslinked chitosan was more than threefold lower at CO_2_ pressure of 60 bar when compare with the hydrogels produced at atmospheric condition. According to Ji, Annabi, Khademhosseini and Dehghani [37] the maximum compression of gas foamed chitosan hydrogel is comparatively greater than the results of our study. This may be due to the high concentration of glutaraldehyde (0.5% *v*/*v*) used for their study. According to the results of Annabi et al. [47] the maximum compression of α-elastin hydrogel produced at dense gas CO_2_ (pressure at 60 bar) was 4.3 ± 1.4 kPa; slightly lower than the results of our study, which is due to the nature of biopolymer used.

### 3.2. FTIR Analysis

**Figure 2:**
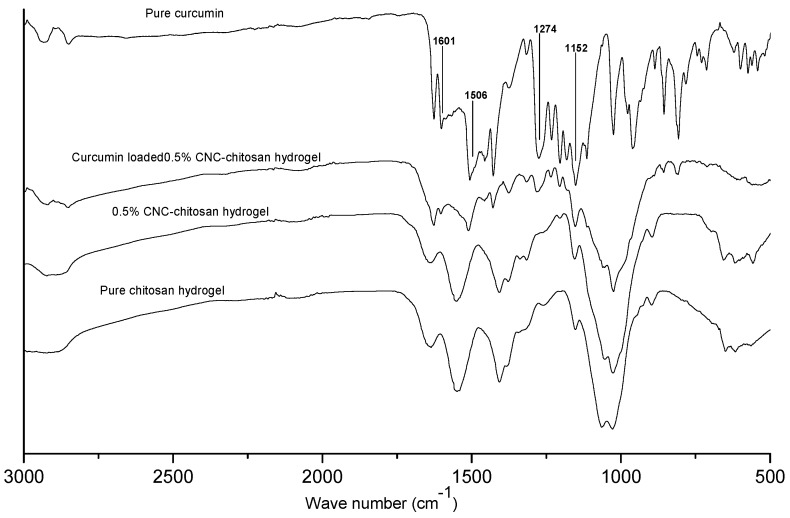
FTIR spectra of pure chitosan hydrogel, 0.5% CNC–chitosan hydrogel, curcumin loaded 0.5% CNC–chitosan hydrogel and pure curcumin.

Figure 2 shows the FTIR spectra of pure chitosan hydrogel, 0.5% CNC–chitosan hydrogel, drug loaded 0.5% CNC–chitosan hydrogel and pure curcumin. Results of our previous study showed that the FTIR spectra of CNC and chitosan are fairly similar due to the chemical similarity between chitosan and cellulose [5]. In the FTIR spectrum of the CNC–chitosan hydrogel, peaks representing both the chitosan hydrogel and CNC were observed [5]. No change or new peak appeared in the spectrum of 0.5% CNC–chitosan hydrogel, thereby indicating that the cellulose nanoparticles were physically added to chitosan hydrogel. The spectrum of curcumin showed characteristic peaks at 1601, 1506, 1274, and 1152 cm^–1^, which corresponded to the stretching vibrations of the benzene ring, C=C vibrations, aromatic C–O stretching, and C–O–C stretching modes, respectively [48,49]. Those characteristic peaks are not shifted significantly in curcumin loaded 0.5% CNC–chitosan hydrogel revealed that there is no interaction between drug and ingredients present in hydrogels. Thus, there is no change in the chemical composition of the drug after the loading process. However, the intensity of the bands in curcumin and curcumin-loaded hydrogel are different. The decrease in peak intensity is due to lower concentration of curcumin in the hydrogel as its concentration was not 100%. Hence, FTIR analysis provides significant evidence for the presence of curcumin in the drug-loaded hydrogel.

### 3.3. Morphology Studies

**Figure 3:**
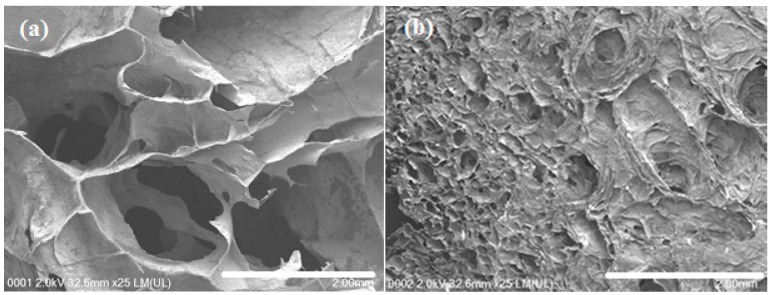

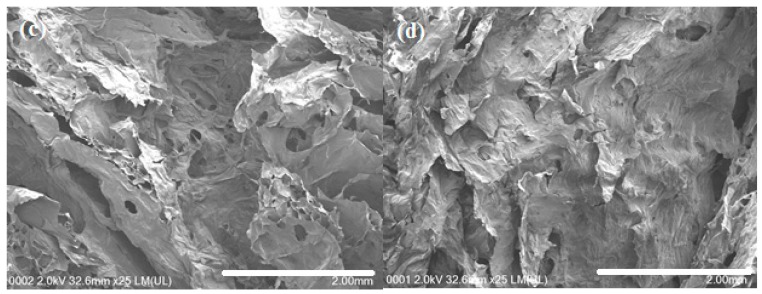
FESEM images for the cross section of: (**a**) gas foamed hydrogel; (**b**) hydrogel formed at atmospheric condition; (**c**) gas foamed hydrogel after immersed in SGF; and (**d**) hydrogel formed at atmospheric condition after immersed in SGF.

Prior to FESEM observation, hydrogels were freeze-dried to remove water to avoid disturbing the morphology of hydrogels. After freeze-drying, hydrogels exhibited a porous network structure (as shown in Figure 3). After the gas foaming (Figure 3a), the pore size significantly increased with the formation of widely interconnected porous structure. Gas foamed hydrogel exhibited rough cell wall structure which exposed more surface area to the drug molecules. Pore sizes of the hydrogel formed at atmospheric pressure (Figure 3b) was around 100 µm and it was more than 10 fold higher in gas foamed hydrogel (at 50 bar)_._

Due to sudden volume expansion, the original morphological features changed in the swollen hydrogel in the drug diffusion medium. In the gas foamed hydrogel, the large pore structure disappeared and small interconnected pores formed after the immersion in SGF (as shown in Figure 3c). On the other hand, the initial porous structure of the hydrogel prepared at atmospheric condition was changed and formed a nonporous structure (Figure 3d). It is clear that the interconnected pores in the swollen state of gas foamed hydrogel promote the swellability, drug loading efficiency, and release as compared to the hydrogel formed at atmospheric condition.

### 3.4. Swelling Ratio

**Figure 4:**
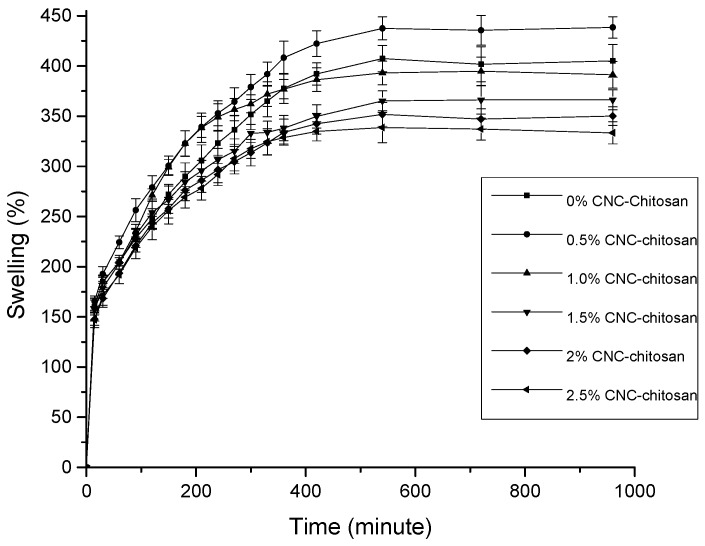
Equilibrium swelling ratio of hydrogels with varying CNC.

Preliminary swelling study is the indicative of the release mechanism of the drug from the swollen polymeric hydrogel [50]. In this study, swelling experiments were carried out in distilled water for chitosan hydrogels prepared with different percentage of CNCs. As shown in Figure 4, swelling increases with time, first rapidly and then slowly, reaching a plateau. All the hydrogel formulations showed more than 100% swelling ratios within first 15 min. In our previous study, we found that the pore sizes of these hydrogels are in the range of several hundred micrometers. According to Ganji et al. [51] the hydrogels possessing pore sizes of several hundred micrometers are classified as super-porous hydrogels (SPHs) which act as a capillary system causing a rapid water uptake into the porous structure. Such a fast swelling is because of absorption of water by capillary force rather than by simple absorption.

After the rapid swelling stage, the swelling ratio increased slowly to reach an equilibrium state. During the process of hydrogel swelling, against the favorable osmotic force, there is an opposite elasticity force, which balances the stretching of the network and prevents its deformation. At equilibrium, elasticity and osmotic forces are balanced and prevent additional swelling [51,52].

Referring to our previous study, all the hydrogels showed highest swelling ratio at acidic medium (pH 4.01). Results of the swelling test of this study showed that the hydrogels swelled more in distilled water than those swelled in buffer solutions (pH 4.01, pH 7 and pH 10.01). Annabi, Mithieux, Weiss and Dehghani [47] described that the reason for lowering of swelling ratio of elastin hydrogels in buffer solutions is due to the presence of salt in buffer solutions resulting in a contraction of the material due to water expulsion.

Out of all types of hydrogels, the 0.5% CNC–chitosan hydrogel showed the highest equilibrium swelling ratio (438% ± 11%), followed by pure chitosan hydrogel (407% ± 13%) and then the 1.0% CNC–chitosan hydrogel (395% ± 14%). Initial swelling is because of water molecules forming hydrogen bonds with hydrophilic functional groups (amine (–NH_2_) and hydroxyl groups (–OH)) present in the chitosan chains. More water molecules then orientate around the bound water to form cage like structures. Finally, excess water enters freely into the hydrogel network resulting in more swelling [53]. All CNC–chitosan hydrogels were expected to show increased swelling ratios due to the hydrophilic property of the CNC. However, our results revealed that the 0.5% CNC–chitosan hydrogel achieved the highest equilibrium swelling ratio. Swelling ratio decreased with further increasing of CNC. Previous studies have described that the decrease of swelling occurred with increase of CNC in the hydrogel is due to the filling up of the free space of hydrogel by CNCs [54].

**Figure 5:**
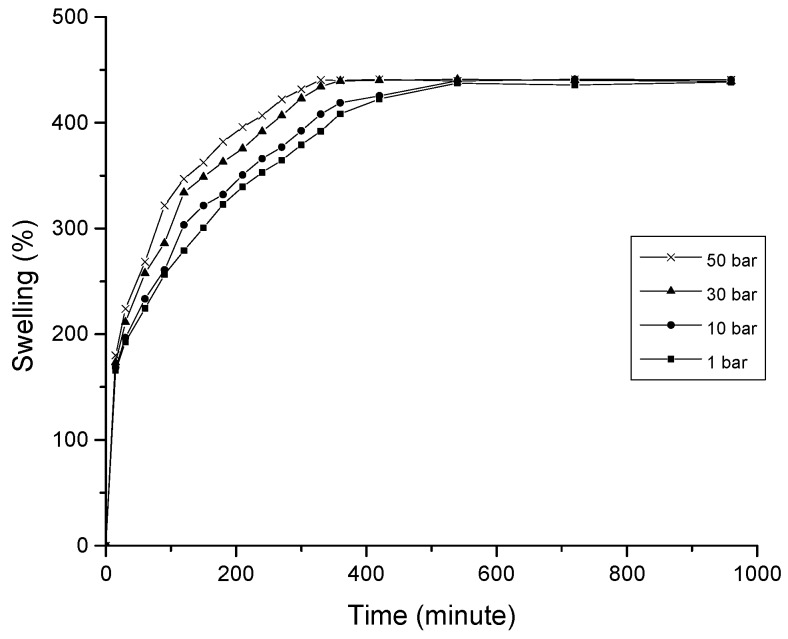
Equilibrium swelling ratio of hydrogels formed at different pressure conditions.

The 0.5 CNC–chitosan hydrogel was used for the CO_2_ gas foaming process as it showed the highest swelling properties in previous swelling studies. As shown in Figure 5, equilibrium swelling ratio of gas foamed hydrogel was studied with varying the CO_2_ pressure of gas foaming process (10, 30 and 50 bar). The experiments were repeated three times and average values were taken. Compared with the hydrogels formed at atmospheric condition, the gas foamed hydrogels swelled faster and reached to the equilibrium stage in a short period of time (around 350 min). However, the maximum swelling ratio of each hydrogel was not varied with the processing pressure. Rapid swelling is due to the formation of larger and interconnected pore network of gas foamed super porous hydrogel (as shown in Figure 3). Open capillary channels in super porous hydrogels absorb water by capillary force rather than by simple absorption resulting in faster swelling behavior [55].

### 3.5. Drug Encapsulation Efficiency

**Figure 6:**
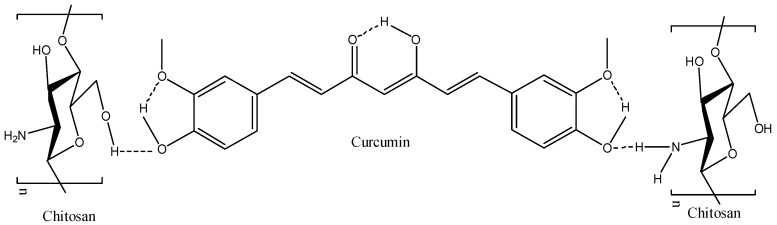
Probable interactions between curcumin and chitosan molecules.

For the curcumin molecule, intramolecular hydrogen bond is the primary interaction which consist of two hydroxyl groups (–OH) in the benzene ring and the hydroxyl near keto group (C=O) [56]. Probable interactions between curcumin and chitosan molecules are shown in Figure 6. The oxygen of hydroxyl on the benzene ring is an important binding site for chitosan molecules. One hydrogen bond can be formed by free hydroxyl groups of glucosamine and the other can be formed by the free amino group of glucosamine. The modeling studies of Liu et al. [57] showed that the lower total energy of curcumin loaded chitosan compared to the total energy of individual molecules proved the possibility of these interactions in the drug loaded system. Thus, it is possible that an interaction will occur between drug and polymer molecules through hydrogen bonding. Results from our previous study showed that the degree of cross linking of chitosan hydrogel was 83.6%. This reveals that more amine groups present in chitosan backbone are occupied by the crosslinking and formation of Schiff base. Therefore, it will limit the availability of free amine groups to bind with drug molecules.

From the data tabulated in Table 1, the maximum drug loading efficiency occurred for the 0.5% CNC–chitosan hydrogel. This result was followed by chitosan hydrogel, the 1% CNC–chitosan hydrogel, and so on. This observed trend was similar to the swelling test pattern of the hydrogels as indicated in Figure 4. When the hydrogel swelling ratio is high, a large amount of drug solution can be absorbed and retained in the hydrogel network, leading to an incremental increase in the drug loading efficiency. Therefore, we can conclude that the efficiency of drug loading in the hydrogel is dependent upon the swelling of the hydrogel in water.

As more CNC was added into the chitosan network, the voids in the chitosan network are gradually filled by CNC, forming a rigid structure and creating a barrier that would prevent the penetration of drug solution in to the hydrogel, which would cause a decrease in the drug loading efficiency.

### 3.6. In Vitro Drug Release

**Figure 7:**
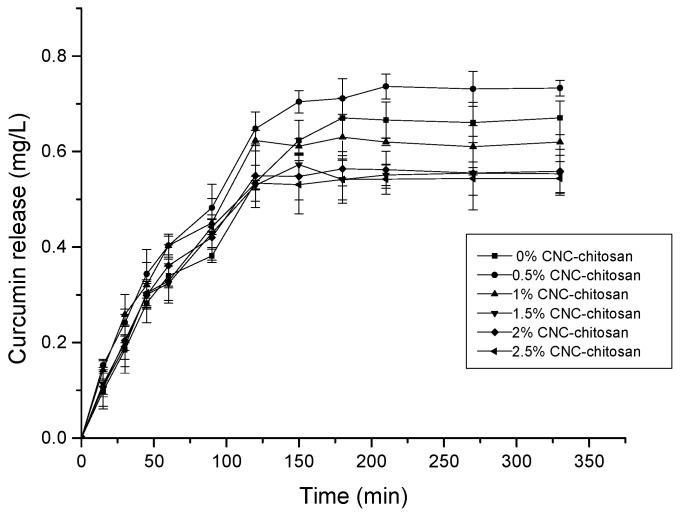
Curcumin release from different types of hydrogels.

The objective of this study is to improve the bioavailability of curcumin to increase absorption from acidic medium in a controlled manner, thereby avoiding wastage of the drug and achieving the desired treatment effect. As shown in Figure 7, the drug release pattern for all hydrogels investigated can be divided in to two phases: an initial burst release and a prolonged diffusion-controlled phase [58]. Rapid drug release in phase one of the test could be due to the presence of the drug on the surface of the hydrogel and the higher drug concentration gradient present at the beginning of the test. This higher concentration gradient could act as the driving force for the drug release from the hydrogel matrix. After the burst release, the releasing rates decline steadily with time, which may be due to the thickness of the hydrogel acting as a diffusion barrier [59].

In general, the rate of drug release from hydrogel matrix is depend on the interaction between the drug and the polymer molecules, the solubility of the drug, and hydrogel swelling in aqueous media [60]. However, in this study, there is no indication for the presence of interaction between hydrogel and drug. Therefore, the rate of drug release in this test depended only on the solubility of the drug and the swelling ratio of the hydrogel in aqueous media. It is clear that the drug release profiles of hydrogels are consistent with the data obtained from the swelling studies. The hydrogel with the higher swelling ratio achieved higher drug release percentages. In this study, the 0.5% CNC–chitosan hydrogel had the highest drug release percentage, whereas the 2.5% CNC–chitosan hydrogel produced the lowest drug release percentage. A lower concentration of curcumin release from these hydrogels is attributable to the fact that curcumin has very poor solubility in water. It has been reported that curcumin has a solubility of around 2.67 µg/mL at pH 7.3 [61].

It is reported that the average gastric emptying times for healthy individuals at 1, 2, and 4 h are >90%, 60% and 10% respectively [62]. Our results showed that all the hydrogels reached to a prolonged diffusion-controlled phase around 120 min. According to the drug release pattern of hydrogels, this drug delivery system can be suggested to improve the bioavailability of curcumin for the absorption from acidic medium during gastric residential time.

### 3.7. Drug Release Kinetics

Curcumin release kinetics was investigated using Ritger–Peppas model (Equation (4)).

(4) $$F_{D}=\frac{M_{t}}{M_{\infty }}=Kt^{n}$$

where *M*_t_/*M*_∞_ is the ratio of curcumin release at time t to the equilibrium swollen state. *K* is the kinetic constant to measure velocity of release and geometrical parameters corresponding to drug–polymer system. *n* is the diffusion exponent related to transport mechanism. If *n* < 0.5, it indicates Fickian diffusion, or drug release that is diffusion-controlled and penetration of solvent into the hydrogel is much faster than the polymer chain relaxation. When 0.5 < *n* < 1, diffusion and release of drug occur in a non-Fickian (anomalous) manner. This means that drug release followed both diffusion and erosion controlled mechanisms [63]. If *n* = 1, it indicates case II transport, where the release rate is constant and controlled by polymer relaxation. The values of *n*, *k* and *R*^2^ of the present study are shown in Table 2. The n values range from 0.61–0.76 when curcumin released in SGF and drug release occurred in non-Fickian (anomalous) manner [64]. If *n* is close to unity, it means solvent penetration controlled by relaxation of polymer chains rather than diffusion [65]. It appears that n value for 0% CNC–chitosan hydrogel is closer to unity when compared with other hydrogels. This may be due to the fact that the 0% CNC–chitosan sample does not contain cellulose nanocrystals within the chitosan matrix. As the hydrogel does not contain the cellulose nanocrystals, there may be some relaxation of chitosan segments within the matrix. In this way, the curcumin release is not totally diffusion controlled but it is partly controlled by chain relaxation process also. On the other hand, there may probably be physical crosslinks between the surface hydroxyls of cellulose crystals and –OH groups of chitosan molecules. The presence of H-bonding interactions between chitosan and cellulose restricts the relaxation of chitosan chains. Therefore, the *n* values for CNC reinforced chitosan hydrogels is lower than the n value of 0% CNC–chitosan hydrogel and drug release depends more on the diffusion rather than relaxation of polymer chains. Furthermore, a significant weight loss was not observed for the hydrogels when immersed in the SGF for 12 h. It may be due to the high degree of cross linking (83.6%) of these hydrogels. This will further clarify that drug release is driven by diffusion driven, rather than erosion of the hydrogel.

The difference in *k* values indicates the difference in physical properties of hydrogel and difference in drug polymer interaction. In addition, the *k* values are smaller due to the less interaction between drug and polymers. In this study, Ritger–Peppas model was used to analyze the drug release kinetics. By using this model, regression coefficient (*R*^2^) was found very close to unity, and it suggested the release data best fit to Ritger–Peppas model.

**Figure 8:**
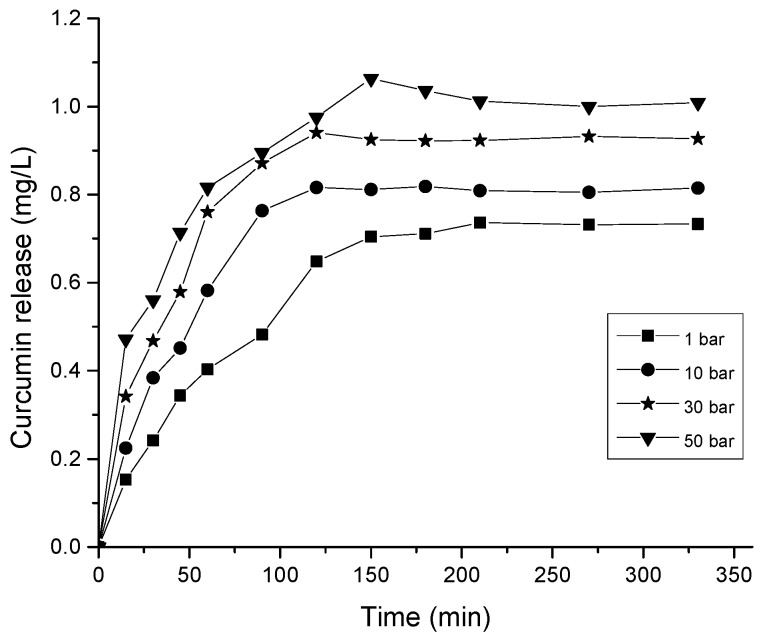
Curcumin release from hydrogel formed at different pressure conditions.

The release of the drug is usually controlled by diffusion and so the release profile can be easily modified by changing the material properties such as pore size or the overall pore surface area, pore connectivity and pore geometry [66]. Larger pore size is suitable to load a high dose of drug molecules [67]. Due to the large pore size of hydrogels, a rapid initial burst release is typically observed. In this study, large-scale macroporous hydrogel with wide interconnected pores and large accessible surface area was obtained with using carbon dioxide gas foaming process. The 0.5% CNC–chitosan hydrogel was used for the gas foaming as it showed the highest swelling ratio and maximum drug release in the previous experiments. As shown in Figure 3, the pore size of hydrogel increased by more than tenfold after the gas foaming process. The increased pore size and pore interconnectivity act as a capillary system causing a rapid diffusion of drug solution through the hydrogel matrix. The drug encapsulation efficiency increased from 41% to 50% with the gas foaming of 0.5% CNC–chitosan hydrogel at 50 bar (as shown in Table 3). In addition, a rapid release of drug and high amount of drug release was observed in the gas foamed hydrogels as were typical of diffusion- controlled systems. As shown in Figure 8, the drug release was increased to 1.06 mg/L in the gas foamed 0.5% CNC–chitosan hydrogel at 50 bar, when compared to the drug release (0.74 mg/L) of 0.5% CNC–chitosan hydrogel formed at atmospheric condition.

The three basic steps in the gas foaming process are: (1) plasticization due to CO_2_ diffusion into the polymer matrix at high pressure; (2) nucleation due to supersaturated gas and depressurization; and (3) gas bubbles growth due to the gas diffusion from the surrounding polymer [68]. Skin layer formation and poor pore interconnectivity are common issues in porous fabrication technique. However, these can be overcome by fabrication of polymer matrices using gas foaming method [69,70]. By tuning the process conditions such as operating pressure, depressurization rate and temperature, the final structure of the product can be modified as required. The results of the previous studies showed that the solubility of CO_2_ dramatically increases as the pressure is increased (0.077 mol CO_2_/kg H_2_O solubility at 1 bar increases to around 30-fold at 50 bar). On the other hand, the solubility of CO_2_ is decreased by increasing the temperature at each pressure [46].

In this study, we fabricated the 0.5% CNC–chitosan hydrogel using high pressure CO_2_ at 10, 30 and 50 bar in room temperature for two days. The operating temperature was not increased (maintained at room temperature) due to the liquefaction of hydrogel at elevated temperatures. Slow depressurization (1 bar/min) was used as it helps to obtain large pore sizes in the hydrogel matrix.

### 3.8. Drug Activity

**Figure 9:**
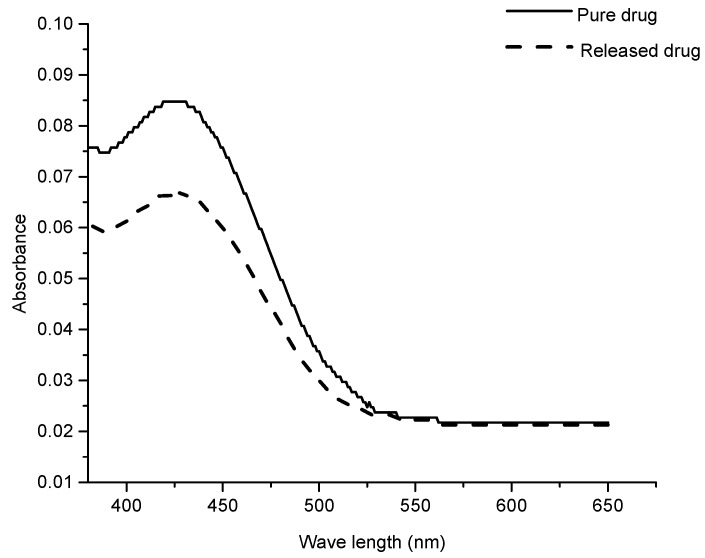
Chemical activity of pure curcumin and curcumin after release.

After releasing, the chemical reactivity and biological activity of the drug are the most critical parameters in drug delivery systems [71]. For some drug delivery systems, drugs deteriorate due to the denaturation reactions with carrier and cause some detrimental effects after release. In order to prevent this the carrier should not interact with the drug and able to be delivered into the body without any chemical transformation. In this study, the activity of curcumin before loading and after release was observed by comparison of UV visible spectra. The spectra of pure curcumin and in the release medium (SGF) were recorded. As shown in Figure 9, a significant difference was not observed for both spectra. This shows that the drug retained its chemical structure after release from the hydrogel.

### 3.9. In Vitro Degradation

**Figure 10:**
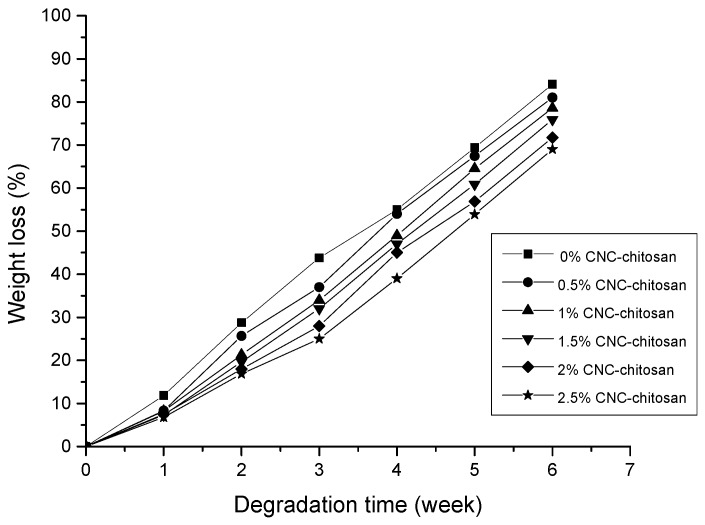
In vitro degradation study of the hydrogels.

For oral drug administration, it is important to study the degradation of hydrogel in conditions similar to living cells over long period of time. Degradation studies were carried out in PBS buffer at 37 °C for six weeks. The results of this study are presented in Figure 10. The hydrogels showed degradation due to the presence of −OH and −NH_2_ groups in the chitosan backbone. These groups have the ability to interact with water. The hydrogel matrix became loose due to swelling property. Gels with low CNC content had greater ability to absorb the solvent and high swelling ability due to the presence of more void space in the hydrogel and loose network [71]. On the other hand, there may be physical crosslinks between the surface hydroxyls of cellulose crystals and –OH groups of chitosan molecules. The presence of H-bonding interactions between chitosan and cellulose restricts the relaxation of chitosan segments within the matrix. Besides that, the crystallinity is an important factor that influences the degradation of hydrogel due to the different chain packing arrangements of each polymer. Amorphous structure allows more water to penetrate in to its polymer matrix resulting faster degradation rates. In the crystalline regions, polymer chains are closely packed and resist the penetration of water molecules within the regions. CNCs are high crystalline particles with highly ordered and closely packed chains. The X-ray diffraction patters of our previous study also indicated that the addition of CNCs will induce a combination of crystalline and amorphous regions in the nanocelulose reinforced chitosan hydrogel. Several studies have also demonstrated that a small amount of CNC could lead to a remarkable decrease in the degradation rate of the composite matrix [72,73]. According to the results, 2.5% CNC–chitosan showed 69% weight loss as it contained high amount of CNCs, while chitosan hydrogel lost its 84% weight because it does not contain CNCs.

## 4. Conclusions

In this study, CNC reinforced chitosan hydrogels were synthesized using chemical crosslinking method. It was observed that compression strength of chitosan hydrogel increased from 25.9 ± 1 kPa to 38.4 ± 1 kPa with the introduction of 0.5% cellulose nanocrystals. Maximum compression of hydrogel decreased with increasing the pressure of gas foaming process due to the formation of large pores and widely interconnected pore structures. FESEM images showed that the pores of the hydrogel are around hundred micrometers and it increased more than tenfold after the gas foaming process (at 50 bar). Further, it showed a highly interconnected open 3-D pore network and rough cell wall structures. Maximum swelling ratio was obtained for 0.5% CNC–chitosan hydrogel and it decreased with increasing the CNC content. This may be due to the filling up the free space of hydrogel with CNCs as indicated by previous studies. Gas foamed hydrogels swelled faster than the hydrogels prepared at atmospheric condition. Results revealed that drug loading efficiency decreased with increasing the percentage of CNC in the hydrogel. Higher drug loading ability was observed in gas foamed hydrogel when compared to hydrogel formed at atmospheric condition. The drug encapsulation efficiency of gas foamed hydrogels at 10, 30 and 50 bar is 43% ± 0.7%, 45% ± 0.9% and 50% ± 2.0%, respectively, and it is 41% ± 2.4% for the hydrogel formed at atmospheric condition. The in vitro curcumin release studies were investigated in SGF. The drug release profiles of hydrogels were consistent with the data obtained from the swelling studies. Highest drug release was indicated by 0.5% CNC–chitosan hydrogel (0.74 ± 0.03 mg/L) and it was lowest in 2.5% CNC–chitosan hydrogel (0.54 ± 0.04 mg/L). The kinetic parameters indicated that the drug diffusion through the hydrogel were in non-Fickian (anomalous) manner. The amount of drug release increased and faster release of drug was observed in the gas foamed hydrogel compared to the hydrogel formed at atmospheric condition. Curcumin retained its structural integrity after release, which is critical requirement for preserving drug activity. In vitro degradation of hydrogels was found dependent on the swelling ratio and the amount of CNC of the hydrogel. Because the pH sensitive CNC-reinforced gas foamed hydrogel exhibited efficient drug carrier properties, it can be suggested as promising candidate for stomach specific drug delivery.

## Figures and Tables

**Table 1:** Encapsulation efficiency of curcumin for different hydrogels.

Hydrogel	Encapsulation Efficiency (%)
0% CNC–chitosan	37 ± 0.8
0.5% CNC–chitosan	41 ± 2.4
1% CNC–chitosan	36 ± 1.8
1.5% CNC–chitosan	34 ± 0.5
2% CNC–chitosan	33 ± 1.9
2.5% CNC–chitosan	30 ± 2.8

**Table 2:** Release kinetics of hydrogel in SGF.

Hydrogel	*n*	*R*^2^	*K*
0% CNC–chitosan	0.76	0.98	0.46
0.5% CNC–chitosan	0.61	0.98	0.52
1% CNC–chitosan	0.68	0.98	0.60
1.5% CNC–chitosan	0.70	0.99	0.57
2% CNC–chitosan	0.67	0.96	0.57
2.5% CNC–chitosan	0.66	0.96	0.57

**Table 3:** Encapsulation efficiency of curcumin for hydrogels formed at different pressure conditions.

Pressure of Hydrogel Formation	Encapsulation Efficiency (%)
1 bar	41 ± 2.4
10 bar	43 ± 0.7
30 bar	45 ± 0.9
50 bar	50 ± 2.0

## References

[1] Mahkam M. (2007). New pH-sensitive glycopolymers for colon-specific drug delivery. Drug Deliv..

[2] Shen X., Shamshina J.L., Berton P., Bandomir J., Wang H., Gurau G., Rogers R.D. (2015). Comparison of hydrogels prepared with ionic-liquid-isolated vs commercial chitin and cellulose. ACS Sustain. Chem. Eng..

[3] Rubentheren V., Ward T.A., Chee C.Y., Tang C.K. (2015). Processing and analysis of chitosan nanocomposites reinforced with chitin whiskers and tannic acid as a crosslinker. Carbohydr. Polym..

[4] Szymańska E., Winnicka K. (2015). Stability of chitosan—A challenge for pharmaceutical and biomedical applications. Mar. Drugs.

[5] Gunathilake T.M.S.U., Ching Y.C., Chuah C.H. Synthesis and characterization of nanocellulose reinforced semi-interpenetrating polymer network of chitosan hydrogel. Cellulose.

[6] Dubey J., Verma A., Verma N. (2015). Evaluation of chitosan based polymeric matrices for sustained stomach specific delivery of propranolol hydrochloride. Indian J. Mater. Sci..

[7] Majithiya R.J., Murthy R.S. (2005). Chitosan-based mucoadhesive microspheres of clarithromycin as a delivery system for antibiotic to stomach. Curr. Drug Deliv..

[8] Patel V.R., Amiji M.M. (1996). Preparation and characterization of freeze-dried chitosan-poly(ethylene oxide) hydrogels for site-specific antibiotic delivery in the stomach. Pharm. Res..

[9] Hejazi R., Amiji M. (2002). Stomach-specific anti-*H. pylori* therapy. I: Preparation and characterization of tetracyline-loaded chitosan microspheres. Int. J. Pharm..

[10] Ali M.S., Pandit V., Jain M., Dhar K.L. (2014). Mucoadhesive microparticulate drug delivery system of curcumin against *Helicobacter pylori* infection: Design, development and optimization. J. Adv. Pharm. Technol. Res..

[11] Thakur A., Monga S., Wanchoo R. (2014). Sorption and drug release studies from semi-interpenetrating polymer networks of chitosan and xanthan gum. Chem. Biochem. Eng. Q..

[12] Wen Y., Li F., Li C., Yin Y., Li J. (2017). High mechanical strength chitosan-based hydrogels cross-linked with poly(ethylene glycol)/polycaprolactone micelles for the controlled release of drugs/growth factors. J. Mater. Chem. B.

[13] Amin S., Rajabnezhad S., Kohli K. (2009). Hydrogels as potential drug delivery systems. Sci. Res. Essays.

[14] Gupta G., Singh A. (2012). A short review on stomach specific drug delivery system. Int. J. PharmTech Res..

[15] Mastropietro D.J., Omidian H., Park K. (2012). Drug delivery applications for superporous hydrogels. Expert Opin. Drug Deliv..

[16] Chavda H., Patel C. (2011). Effect of crosslinker concentration on characteristics of superporous hydrogel. Int. J. Pharm. Investig..

[17] Chang C.W., van Spreeuwel A., Zhang C., Varghese S. (2010). PEG/clay nanocomposite hydrogel: A mechanically robust tissue engineering scaffold. Soft Matter.

[18] Park M., Lee D., Hyun J. (2015). Nanocellulose-alginate hydrogel for cell encapsulation. Carbohydr. Polym..

[19] Mohd A.C.M., Ching Y.C., Luqman C.A., Poh S.C., Chuah C.H. (2016). Review of bionanocomposite coating films and their applications. Polymers.

[20] Ching Y.C., Ershad A., Luqman C.A., Choo K.W., Yong C.K., Sabariah J.J., Chuah C.H., Liou N.S. (2016). Rheological properties of cellulose nanocrystal-embedded polymer composites: A review. Cellulose.

[21] Choo K.W., Ching Y.C., Chuah C.H., Sabariah J., Liou N.S. (2016). Preparation and characterization of polyvinyl alcohol-chitosan composite films reinforced with cellulose nanofiber. Materials.

[22] Yang J., Han C.R., Duan J.F., Ma M.G., Zhang X.M., Xu F., Sun R.C., Xie X.M. (2012). Studies on the properties and formation mechanism of flexible nanocomposite hydrogels from cellulose nanocrystals and poly(acrylic acid). J. Mater. Chem..

[23] Yang X., Bakaic E., Hoare T., Cranston E.D. (2013). Injectable polysaccharide hydrogels reinforced with cellulose nanocrystals: Morphology, rheology, degradation, and cytotoxicity. Biomacromolecules.

[24] Ahmed E.M. (2015). Hydrogel: Preparation, characterization, and applications: A review. J. Adv. Res..

[25] Das N. (2013). Preparation methods and properties of hydrogel: A review. Int. J. Pharm. Pharm. Sci..

[26] Prasad S., Tyagi A.K., Aggarwal B.B. (2014). Recent developments in delivery, bioavailability, absorption and metabolism of curcumin: The golden pigment from golden spice. Cancer Res. Treat..

[27] Mahattanadul S. (2016). Floating gellan gum-based in situ gels containing curcumin for specific delivery to the stomach. Thai J. Pharm. Sci..

[28] Ridhima D., Shweta P., Upendra K. (2012). Formulation and evaluation of floating microspheres of curcumin. Int. J. Pharm. Pharm. Sci.

[29] Treesinchai S., Puttipipatkhachorn S., Pitaksuteepong T., Sungthongjeen S. (2016). Development of curcumin floating tablets based on low density foam powder. Asian J. Pharm. Sci..

[30] Shivashankar M., Mandal B.K. (2012). A review on interpenetrating polymer network. Int. J. Phram. Phram. Sci.

[31] Guo D., Xie G., Luo J. (2013). Mechanical properties of nanoparticles: Basics and applications. J. Phys. D.

[32] Han K.S., Song J.E., Tripathy N., Kim H., Moon B.M., Park C.H., Khang G. (2015). Effect of pore sizes of silk scaffolds for cartilage tissue engineering. Macromol. Res..

[33] Chiu Y.C., Kocagöz S., Larson J.C., Brey E.M. (2013). Evaluation of physical and mechanical properties of porous poly(ethylene glycol)-*co*-(l-lactic acid) hydrogels during degradation. PLoS ONE.

[34] Wang H., Roman M. (2011). Formation and properties of chitosan-cellulose nanocrystal polyelectrolyte—Macroion complexes for drug delivery applications. Biomacromolecules.

[35] Akhlaghi S.P., Berry R.C., Tam K.C. (2013). Surface modification of cellulose nanocrystal with chitosan oligosaccharide for drug delivery applications. Cellulose.

[36] Ng T.S., Ching Y.C., Awanis N., Ishenny N., Rahman M.R. (2014). Effect of bleaching condition on thermal properties and UV-transmittance of PVA/cellulose biocomposites. Mater. Res. Innov..

[37] Goh K.Y., Ching Y.C., Chuah C.H., Luqman C.A., Liou N.S. (2016). Individualization of microfibrillated celluloses from oil palm empty fruit bunch: Comparative studies between acid hydrolysis and ammonium persulfate oxidation. Cellulose.

[38] Ching Y.C., Rahman A., Ching K.Y., Sukiman N.L., Cheng H.C. (2015). Preparation and characterization of polyvinyl alcohol-based composite reinforced with nanocellulose and nanosilica. BioResources.

[39] Rubentheren V., Thomas A.W., Ching Y.C., Praveena N., Erfan S., Christopher F. (2016). Effects of heat treatment on chitosan nanocomposite film reinforced with nanocrystalline cellulose and tannic acid. Carbohydr. Polym..

[40] Rubentheren V., Ward T.A., Chee C.Y., Nair P. (2015). Physical and chemical reinforcement of chitosan film using nanocrystalline cellulose and tannic acid. Cellulose.

[41] Mak A.F.T., Sun S., Hilgers P., Riechers A., König B. (2008). Chapter 18 intelligent chitosan-based hydrogels as multifunctional materials. Intelligent Materials.

[42] Giri T.K., Thakur A., Alexander A., Badwaik H., Tripathi D.K. (2012). Modified chitosan hydrogels as drug delivery and tissue engineering systems: Present status and applications. Acta Pharm. Sin. B.

[43] Berger J., Reist M., Mayer J.M., Felt O., Peppas N., Gurny R. (2004). Structure and interactions in covalently and ionically crosslinked chitosan hydrogels for biomedical applications. Eur. J. Pharm. Biopharm..

[44] Hooda A. (2012). Gastroretentive drug delivery systems: A review of formulation approaches. Pharm. Innov..

[45] Annabi N., Nichol J.W., Zhong X., Ji C., Koshy S., Khademhosseini A., Dehghani F. (2010). Controlling the porosity and microarchitecture of hydrogels for tissue engineering. Tissue Eng. Part B.

[46] Ji C., Annabi N., Khademhosseini A., Dehghani F. (2011). Fabrication of porous chitosan scaffolds for soft tissue engineering using dense gas CO_2_. Acta Biomater..

[47] Annabi N., Mithieux S.M., Weiss A.S., Dehghani F. (2009). The fabrication of elastin-based hydrogels using high pressure CO_2_. Biomaterials.

[48] Zhao Z., Xie M., Li Y., Chen A., Li G., Zhang J., Hu H., Wang X., Li S. (2015). Formation of curcumin nanoparticles via solution-enhanced dispersion by supercritical CO_2_. Int. J. Nanomed..

[49] Pawar H., Karde M., Mundle N., Jadhav P., Mehra K. (2014). Phytochemical evaluation and curcumin content determination of turmeric rhizomes collected from bhandara district of maharashtra (India). Med. Chem..

[50] Sim S., Figueiras A., Veiga F. (2012). Modular hydrogels for drug delivery. J. Biomater. Nanobiotechnol..

[51] Ganji F., Vasheghani-Farahani S., Vasheghani-Farahani E. (2010). Theoretical description of hydrogel swelling: A review. Iran Polym. J..

[52] Saber-Samandari S., Gazi M. (2015). Pullulan based porous semi-IPN hydrogel: Synthesis, characterization and its application in the removal of mercury from aqueous solution. J. Taiwan Inst. Chem. E.

[53] Khurma J.R., Rohindra D.R., Nand A.V. (2006). Synthesis and properties of hydrogels based on chitosan and poly(vinyl alcohol) crosslinked by genipin. J. Macromol. Sci. Part A.

[54] Ooi S.Y., Ahmad I., Amin M.C.I.M. (2016). Cellulose nanocrystals extracted from rice husks as a reinforcing material in gelatin hydrogels for use in controlled drug delivery systems. Ind. Crop. Prod..

[55] Gemeinhart R.A., Park H., Park K. (2000). Pore structure of superporous hydrogels. Polym. Adv.Technol..

[56] Wegiel L.A., Zhao Y., Mauer L.J., Edgar K.J., Taylor L.S. (2014). Curcumin amorphous solid dispersions: The influence of intra and intermolecular bonding on physical stability. Pharm. Dev. Technol..

[57] Liu Y., Cai Y., Jiang X., Wu J., Le X. (2016). Molecular interactions, characterization and antimicrobial activity of curcumin–chitosan blend films. Food Hydrocoll..

[58] Huang X., Brazel C.S. (2001). On the importance and mechanisms of burst release in matrix-controlled drug delivery systems. J. Control. Releas..

[59] Fu Y., Kao W.J. (2010). Drug release kinetics and transport mechanisms of non-degradable and degradable polymeric delivery systems. Expert Opin. Drug Deliv..

[60] Kim S.W., Bae Y.H., Okano T. (1992). Hydrogels: Swelling, drug loading, and release. Pharm. Res..

[61] Bajpai S., Chand N., Ahuja S. (2015). Investigation of curcumin release from chitosan/cellulose micro crystals (CMC) antimicrobial films. Int. Biol. Macromol..

[62] Jobe B.A., Richter J.E., Hoppo T., Peters J.H., Bell R., Dengler W.C., DeVault K., Fass R., Gyawali C.P., Kahrilas P.J. (2013). Preoperative diagnostic workup before antireflux surgery: An evidence and experience-based consensus of the esophageal diagnostic advisory panel. J. Am. Coll. Surg..

[63] Emami J., Tajeddin M., Ahmadi F. (2010). Preparation and in vitro evaluation of sustained-release matrix tablets of flutamide using synthetic and naturally occurring polymers. Iran. J. Pharm. Res..

[64] Chime S., Onunkwo G., Onyishi I. (2013). Kinetics and mechanisms of drug release from swellable and non swellable matrices: A review. Res. J. Pharm. Biol. Chem. Sci..

[65] Agnihotri S.A., Aminabhavi T.M. (2006). Novel interpenetrating network chitosan-poly(ethylene oxide-*g*-acrylamide) hydrogel microspheres for the controlled release of capecitabine. Int. J. Pharm..

[66] Gonzalez G., Sagarzazu A., Zoltan T. (2013). Infuence of microstructure in drug release behavior of silica nanocapsules. J. Drug Deliv..

[67] Kwon S., Singh R.K., Perez R.A., Neel E.A.A., Kim H.W., Chrzanowski W. (2013). Silica-based mesoporous nanoparticles for controlled drug delivery. J. Tissue Eng..

[68] Annabi N., Fathi A., Mithieux S.M., Weiss A.S., Dehghani F. (2011). Fabrication of porous PCL/elastin composite scaffolds for tissue engineering applications. J. Supercrit. Fluids.

[69] Dehghani F., Annabi N. (2011). Engineering porous scaffolds using gas-based techniques. Curr. Opin. Biotechnol..

[70] Sampath U.G., Ching Y.C., Chuah C.H., Sabariah J.J., Lin P.C. (2016). Fabrication of porous materials from natural/synthetic biopolymers and their composites. Materials.

[71] Bashir S., Teo Y.Y., Ramesh S., Ramesh K. (2016). Synthesis, characterization, properties of *n*-succinyl chitosan-*g*-poly(methacrylic acid) hydrogels and in vitro release of theophylline. Polymer.

[72] Abou-Zeid R.E., Hassan E.A., Bettaieb F., Khiari R., Hassan M.L. (2015). Use of cellulose and oxidized cellulose nanocrystals from olive stones in chitosan bionanocomposites. J. Nanomater..

[73] De Paula E.L., Mano V., Duek E.A.R., Pereira F.V. (2015). Hydrolytic degradation behavior of PLLA nanocomposites reinforced with modified cellulose nanocrystals. Química Nova.

